# A real‐life pilot study of the clinical application of pharmacogenomics testing on saliva in epilepsy

**DOI:** 10.1002/epi4.12717

**Published:** 2023-05-15

**Authors:** Antonella Riva, Roberta Roberti, Gianluca D'Onofrio, Maria Stella Vari, Elisabetta Amadori, Valentina De Giorgis, Caterina Cerminara, Nicola Specchio, Nicola Pietrafusa, Mario Tombini, Giovanni Assenza, Silvia Cappanera, Carla Marini, Paolo Rasmini, Pierangelo Veggiotti, Federico Zara, Emilio Russo, Pasquale Striano

**Affiliations:** ^1^ Department of Neurosciences Rehabilitation, Ophthalmology, Genetics, Maternal and Child Health (DINOGMI) University of Genoa Genoa Italy; ^2^ Pediatric Neurology and Muscular Diseases Unit IRCCS Istituto “Giannina Gaslini” Genoa Italy; ^3^ Science of Health Department University Magna Grecia of Catanzaro Catanzaro Italy; ^4^ Department of Child Neurology and Psychiatry IRCCS Mondino Foundation Pavia Italy; ^5^ Pediatric Neurology Unit, Department of Neurosciences Tor Vergata University of Rome Rome Italy; ^6^ Rare and Complex Epilepsy Unit, Department of Neuroscience Bambino Gesù Children's Hospital, IRCCS Rome Italy; ^7^ Unit of Neurology, Neurophysiology, Neurobiology, Department of Medicine University Campus Bio‐Medico Rome Italy; ^8^ Child Neurology and Psychiatric Unit, Pediatric Hospital G. Salesi United Hospitals of Ancona Ancona Italy; ^9^ Child Neuropsychiatry Vercelli Vercelli Italy; ^10^ Unit of Medical Genetics IRCCS Istituto “Giannina Gaslini” Genoa Italy

**Keywords:** antiseizure medications, epilepsy, pharmacogenomics, precision medicine

## Abstract

Response to antiseizure medications (ASMs) can be influenced by several gene polymorphisms, causing either lower efficacy or higher occurrence of adverse drug reactions (ADRs). We investigated the clinical utility of salivary pharmacogenomic testing on epilepsy patients. A commercialized pharmacogenomic salivary test was performed in a cohort of epileptic patients. Genetic variants on five genes (i.e., *CYP1A2*, *CYP2C9*, *CYP2C19*, *EPHX1*, and *ABCB1*) involved in common ASMs metabolism were selected. Twenty‐one individuals (median age [Q_1_–Q_3_]: 15 [6.5–28] years) were enrolled. Six patients harboring the homozygous *1F allele in *CYP1A2* could have reduced chance of response to stiripentol due to fast metabolism. *CYP2C9* had reduced activity in 10 patients (alleles *2 and *3), potentially affecting phenytoin (PHT), phenobarbital (PB), primidone, lacosamide (LCM), and valproic acid metabolism. Seven patients, carrying the *2 allele of *CYP2C19*, had an increased risk of ADRs with clobazam (CLB), PB, PHT, LCM, brivaracetam; while one individual with the *17 allele in heterozygosity reported a CLB fast metabolism. Six patients showed a CC polymorphism of *EPHX1* associated with the impaired efficacy of carbamazepine. *ABCB1* polymorphisms related to drug‐resistance (3435 CC) or drug‐sensitive phenotype (CT or TT) were found in 6 out of 7 patients. Pharmacogenomic testing on saliva proved easy and safe in clinical practice to convey information for the management of epileptic patients, especially those resistant to treatment or sensitive to severe ADRs.

## 1 INTRODUCTION

Despite the availability of more than 20 antiseizure medications (ASMs), up to 35% of epileptic patients are refractory to treatment. Moreover, there is clear evidence of heterogeneous responses to ASMs^1^, ^2^ and one‐third of patients experience adverse drug reactions (ADRs) ranging from mild up to severe.^3^

Antiseizure medications efficacy and ADRs susceptibility vary widely across individuals. Apart from carbamazepine (CBZ) rash and some pharmacokinetic markers, the study of pharmacogenomics in epilepsy has been largely disappointing by date. Pharmacogenomics analyzes the genetic makeup of an individual to predict drug response and efficacy, as well as potential ADRs. The novel genetic techniques, which can analyze a large series of known genes at a reasonable price, are of paramount importance to discovering novel therapeutic avenues.^1^, ^4^ We explored the potential clinical utility and influence on therapeutic strategies of pharmacogenomic testing in a small cohort of patients with epilepsy.

## 2 METHODS

### 2.1 Study design and patients' cohort

This was a multicentric, naturalistic study in Caucasian patients with epilepsy of different etiologies. Patients voluntarily performed a widely commercialized pharmacogenomic test (Neuropharmagen®) carried out on saliva, providing extensive genetic‐based information on drug efficacy, metabolism, and ADRs. The test brings principally pharmacokinetics information on over 50 ASMs, antidepressants, and antipsychotic drugs.^5^

We enrolled patients diagnosed according to the International League Against Epilepsy classification^6^ from seven tertiary epilepsy centers. Demographic and clinical data, including the patients' age and gender, age at epilepsy onset, epilepsy type, any neuropsychiatric comorbidity, failed and current treatments, were collected in a specific form, and descriptive statistical analyses were performed.

### 2.2 Genetic analysis

Patients' saliva sample was collected using Oragene DNA Sample Collection Kit (OG‐510; DNA Genotek Inc., USA). Saliva samples were then shipped to AB‐Biotics (Girona, Spain) laboratory for DNA extraction and analysis. DNA isolation was performed with the Genomic DNA Isolation Kit (Norgen Biotek Corp., Canada). DNA quality and concentration were measured with a Nanodrop 2000 microvolume spectrophotometer (Thermo Fischer Scientific Inc., USA). Single‐nucleotide polymorphism (SNP) genotype was then performed by Golden Gate Technology (Illumina Inc., USA). Data were generated with the BeadXpress Reader (Illumina Inc.) and then analyzed with Genome Studio Data Analysis Software (Illumina Inc.), which performs automated genotype clustering and calling. A sample with a call rate below 98% was discarded. All assays were performed in quadruplicate in a 7500 RT‐PCR System using TaqMan Genotyping Master Mix (Life Technologies Inc., Germany) using the comparative ΔΔCT method. We focused on genetic variants of five selected genes (*CYP1A2*, *CYP2C9*, *CYP2C19*, *EPHX1*, and *ABCB1*) largely involved in ASMs metabolism or transport^7^ that could have significant implications on patients' treatment. The correspondent mutant alleles and related SNPs are reported in Table S1.

### 2.3 Clinical implications' assessment

The impact of the selected SNPs on the response to ASMs has been assessed through a literature search on MEDLINE/PubMed, Scopus, and Web of Science up to September 2022 by combining separately the terms “*CYP1A2*,” “*CYP2C9*,” “*CYP2C19*,” “*EPHX1*,” “*ABCB1*” with the terms “polymorphism” AND “antiepileptic drug*”. Only English‐written papers were included. Literature data were combined with pharmacogenomic results, and the utility of genetic testing was evaluated through a comparison between the suggestions obtained and the previous/current treatments of our cohort.

## 3 RESULTS

### 3.1 Clinical features

Twenty‐one patients (52.4% females) with a mean age of 20 years (age range: 2–60 years; median [Q_1_–Q_3_]: 15 [6.5–28]) were analyzed by the pharmacogenomics panel. This cohort included patients with focal (10 individuals, 47.6%) or generalized (3 individuals, 14.3%) epilepsy and with epileptic encephalopathy (8 individuals, 38.1%). Age at epilepsy diagnosis ranged from 4 months to 51 years (mean: 10 years; median: 4 [1–15]).

In 14 subjects (66.7%), epilepsy was associated with neuropsychiatric comorbidities. Patients had failed up to 9 previous therapeutic attempts (mean: 3.4) and received up to 4 ASMs (mean: 2.5) at the time of pharmacogenomic testing.

### 3.2 Genetic analysis and clinical implications' assessment

Table 1 shows the genotype, phenotype, and the allele frequencies related to the five analyzed genes.

**TABLE 1:** *CYP1A2, CYP2C9*, *CYP2C19*, *EPHX1*, and *ABCB1* genotypes, phenotypes, and frequencies.

Gene	Genotype	Phenotype	Frequency
CYP1A2	*1/*1F	EM	15/21 (71.4%)
	*1F/*1F	FM	6/21 (28.6%)
CYP2C9	*1/*1	EM	11/21 (52.4%)
	*1/*2	IM	5/21 (23.8%)
	*1/*3		4/21 (19%)
	*2/*3	PM	1/21 (4.8%)
CYP2C19	*1/*1	EM	10/21 (47.6%)
	*1/*17		3/21 (14.3%)
	*1/*17	FM	1/21 (4.8%)
	*1/*2	IM	6/21 (28.5%)
	*2/*2	PM	1/21 (4.8%)
*EPHX1*	337T>C (CC)	↓ efficacy CBZ	6/21 (28.5%)
*ABCB1*	3489 + 80C>T (CC)	Drug‐resistant	6/21 (28.5%)
	3489 + 80C>T (CT or TT)	Drug‐sensitive	7/21 (33.3%)

Abbreviations: EM, extensive metabolizer (standard); FM, fast metabolizer; IM, intermediate metabolizer; PM, poor metabolizer.

Colour shade indicate to distinguish the different aplotypes and their effect on cytocromes.

The genotype influence on drug response according to ASMs' pharmacokinetic characteristics is displayed in Table S2.

Six patients with the homozygous *1F allele in *CYP1A2* may have reduced chance of response to stiripentol (STP) due to fast metabolism. Nine CYP2C9 intermediate metabolizers (IM) (*1/*2 and *1/*3) and one poor metabolizer (PM) were at increased risk of ADRs with phenytoin (PHT), phenobarbital (PB), primidone, lacosamide (LCM), and valproic acid (VPA).

The *CYP2C19* allele *2 reduces substrates metabolism both in heterozygosis (*1/*2) and homozygosis (*2/*2) and it was found in 7 individuals (6 IM and 1 PM).

CC polymorphism of *EPHX1* was associated with impaired efficacy of CBZ, and it was genotyped in 6 patients.

*ABCB1* is involved in transport of most of the ASMs. Polymorphism 3435 CC was related to drug resistance, and it was genotyped in 6 individuals, while CT or TT has led to a drug‐sensitive phenotype in 7 patients. Only 2 patients were wild type for all the selected genes.

Antiseizure medication pharmacokinetic and patients' pharmacogenomic data were combined in Table 2 suggesting drugs with a higher risk of ineffectiveness or ADRs according to each patient's genotype.

**TABLE 2:** Personalized drug selection suggestions based on pharmacogenomic data.

	#1	#2	#3	#4	#5	#6	#7	#8	#9	#10	#11	#12	#13	#14	#15	#16	#17	#18	#19	#20	#21
Brivaracetam			§	§		§					§				§		*		§		§
Cannabidiol		*	§	§		§	*	*	*		§				§		*		*	*	§
Cenobamate																					
Carbamazepine					*	*					*			*						*	*
Clobazam		*	§	§		§	*	*	*		§				§		*		*	*	§
Clonazepam		*					*	*	*										*	*	
Eslicarbazepine		*					*	*	*										*	*	
Ethosuximide																					
Everolimus		*					*	*	*										*	*	
Felbamate		*					*	*	*										*	*	
Gabapentin																					
Lacosamide	§	§	§	§		§			§		§	§		§	§	§	*		*	§	§
Lamotrigine		*					*	*	*										*	*	
Levetiracetam		*					*	*	*										*	*	
Oxcarmazepine		*					*	*	*											*	
Perampanel																					
Phenobarbital	§	*		§			*	*	*		§	§		§	§	§			*	*	
Phenytoin	§	*	§	§		§	*	*	*		§	§		§	§	§	*		*	*	§
Pregabalin																					
Primidone	§	*		§			*	*	*		§	§		§	§	§			*	*	
Rufinamide																					
Stiripentol		*	§	§		§	*	*	*		§		*	*	§		*		*	*	*
Tiagabine		*					*	*	*										*	*	
Topiramate		*					*	*	*										*	*	
Valproic acid	§	§		§					§		§	§		§	§	§				§	
Vigabatrin																					
Zonisamine																					

*Note*: Orange = need of monitoring due to: *Reduced chance of optimal response, ^§^Increased risk of adverse events; green = optimal safety and efficacy profile.

Table 3 shows a complete overview of demographic, clinical, and genetic data of the enrolled patients, with clinical implications of genetic testing. In seven patients (33%), the test prescription highlighted the current use of ASMs at increased risk of ADRs. The poor response to previous treatments could be predicted in seven patients (33%), whereas in five patients (23.8%) medications that should be avoided in the future were identified.

**TABLE 3:** Clinical implications and importance of pharmacogenetic testing according to each patient's characteristics.

Patient code	Gender	Age (y)	Diagnosis	Comorbidities	Failed treatments	Current treatment	Genetic variants (phenotypes)	Clinical implications
#1	M	18	FNLE	None	CLB, CBZ	OXC, LCM, PER, LEV	CYP2C9 *1/*2 (IM) CYP2D6 *41/*41 (IM)	Increased risk of AEs with LCM
#2	M	14	FNLE	ID	CLB, PER	TPM, CBZ	CYP1A2 *1F/*1F (FM) CYP2C9 *1/*2 (IM) ABCB1 3489 + 80CC	CBZ is the optimal choice
#3	F	14	EE	ID, psychosis	VGB, NZP, VPA, CLB, TPM, ACTH	ACZ, LTG, ZNS, PER	CYP2C19 *1/*2 (IM)	CLB could have been avoided
#4	F	5	EE	ID	CLB, GVG, LTG, TPM	VPA, RFM, ACZ, NZP	CYP2C9 *1/*2 (IM) CYP2C19 *1/*2 (IM)	CLB could have been avoided
#5	F	9	GGE	ID	ACTH	LTG, VPA	EPHX1 337 CC	CBZ should be avoided
#6	F	7	FNLE	None	PB, LEV, CCS	VPA, TPM, CLB	CYP2C19 *2/*2 (PM) EPHX1 337 CC	Increased risk of AEs with CLB; CBZ should be avoided
#7	M	19	EE	ASD, ID	LEV, ETS, ZNS, FBM, LTG, TPM, PB, CBZ, VGB	VPA, CLB, RFM	CYP1A2 *1F/*1F (FM) ABCB1 3489 + 80CC	Pharmacoresistance could have been predicted
#8	F	30	EE	ID	VPA, ZNS, TPM, CLB, ETS, LTG, FBM, PHT	LEV, OXC, CNZ	CYP1A2 *1F/*1F (FM) CYP2D6 *2/*4 (IM) ABCB1 3489 + 80CC	Pharmacoresistance could have been predicted
#9	M	6	GGE	ASD, ID	VPA, ETS	None	CYP2C9 *1/*3 (IM) ABCB1 3489 + 80CC	VPA could have been avoided
#10	F	8	GGE	None	VPA, CNZ	ETS, LCM	–	None
#11	M	19	EE	ASD, ID	FBM, RFM, LEV, ZNS, CLB	CNZ, NZP, VPA, PGB	CYP2C9 *1/*3 (IM) CYP2C19 *1/*2 (IM) EPHX1 337 CC	Increased risk of AEs with VPA; CLB could have been avoided; CBZ should be avoided
#12	F	20	FLE	None	CLB	LEV, CBZ, LCM	CYP2C9 *1/*3 (IM)	Increased risk of AEs with LCM
#13	M	26	FNLE	Bipolar disorder	CBZ, OXC, VGB, PB, ZNS, TPM, LTG	VPA	CYP1A2 *1F/*1F (FM)	Decreased antipsychotics blood levels
#14	M	5	EE	ID	CNZ, TPM, ETS	LEV, STP, CLB, VPA	CYP1A2 *1F/*1F (FM) CYP2C9 *1/*2 (IM) EPHX1 337 CC	Increased risk of AEs with VPA; CBZ should be avoided
#15	F	43	FNLE	Bipolar disorder	CBZ, OXC, VGB, PB, ZNS, TPM	LTG, VPA, CLB	CYP2C9 *1/*3 (IM) CYP2C19 *1/*2 (IM)	PB could have been avoided; Increased risk of AEs with VPA and CLB
#16	M	2	EE	ID	CLB	VPA, CBZ	CYP2C9 *2/*3 (PM)	Increased risk of AEs with VPA
#17	M	60	FNLE	None	None	LEV, CBZ	CYP2C19 *1/*17 (FM)	CYP2C19 as CLB should be avoided
#18	F	44	FNLE	None	LEV, CBZ	TPM	CYP2D6 *2/*4 (IM)	None
#19	M	6	EE	ID	PB, VPA, CBZ, CNZ, CLB, LEV	LCM, PB, RFM	CYP2C19 *1/*2 (IM) CYP2D6 *4/*4 (PM) ABCB1 3489 + 80CC	CLB could have been avoided; Risk of pharmacoresistance; prefer ASMs which are not ABCB1 substrates
#20	F	15	FNLE	ID, psychosis	CNZ	VPA, CLB	CYP2C9 *1/*2 (IM) EPHX1 337 CC ABCB1 3489 + 80CC	Risk of pharmacoresistance; prefer ASMs which are not ABCB1 substrates
#21	F	51	FLE	No	CBZ, VPA	ZNS	CYP1A2 *1F/*1F (FM) CYP2C19 *1/*2 (IM) CYP2D6 *1/*5 (IM) EPHX1 337 CC	CBZ could have been avoided

Abbreviations: ACTH, adrenocorticotropic hormone; ACZ, acetazolamide; AEs, adverse events; ASD, autism spectrum disorder; ASMs, antiseizure medications; CBZ, carbamazepine; CCS, corticosteroids; CLB, clobazam; CNZ, clonazepam; EE, epileptic encephalopathy; ETS, ethosuximide; FBM, felbamate; FLE, focal lesional epilepsy; FM, fast metabolizer; FNLE, focal nonlesional epilepsy; GGE, genetic generalized epilepsy; ID intellectual disability; IM, intermediate metabolizer; LCM, lacosamide; LEV, levetiracetam; LTG, lamotrigine; NZP, nitrazepam; OXC, oxcarbazepine; PER, perampanel; PB, phenobarbital; PGB, pregabalin; PHT, phenytoin; PM, poor metabolizer; RFM, rufinamide; TPM, topiramate; VPA, valproic acid; VGB, vigabatrin; y, years; ZNS, zonisamide.

Pharmacogenomic test also included analysis of *CYP2B6*, *CYP2D6*, and *CYP3A4* (Table S3), which may influence the metabolism of several antidepressant and antipsychotic drugs. The analysis of these cytochromes was beyond the aim of the current study; however, Table 3 shows the results of clinical interest that could add information to improve the management of patients with psychiatric comorbidities.

## 4 DISCUSSION

This pilot study aimed to explore the “real‐life” impact of pharmacogenomic testing and its potential clinical utility. As variations in drug‐metabolizing genes^8^ are known to have the most clinical impact on ASM therapy, we particularly focused on *CYP1A2*, *CYP2C9*, *CYP2C19*, *EPHX1*, and *ABCB1*.

Among the most extensively studied CYP1A2 polymorphisms, the CYP1A2*1F haplotype influences the inducibility of the enzyme, leading to higher enzyme activity. However, this phenotype effect is observed only in the presence of an inducer (e.g., smoking or heavy coffee consumption),^9^ and other CYP enzymes contribute to STP metabolism, so we considered as “potential” the reduced chance of drug response in our cohort.

Noteworthy, CYP1A2 is largely involved in antipsychotics' metabolism, and we found a CYP1A2*1F/*1F genotype in a patient with bipolar disorder (#13), who could be at increased risk of inadequate response to antipsychotics, potentially improving the management of epilepsy comorbidities.

CYP2C9*1 is designated as the wild‐type allele, whereas CYP2C9*2 and *3 lead to a reduction in functional activity.^10^ CYP2C9 is known to be involved in the metabolism of PHT and polymorphisms resulting in reduced PHT clearance may increase the risk of ADRs.^11^ Likewise, the CYP2C9*2 and *3 alleles were associated with a significant reduction in VPA metabolism, especially in children, in whom CYP2C9 represents the main VPA metabolic route.^12^

Nearly half of our patients carried the *1/*2 and *1/*3 genotypes (intermediate metabolizer phenotype) and *2/*3 (poor metabolizer phenotype). The presence of these genotypes reduces the chances of optimal response to PHT and VPA and there is a need to monitor dosage.

*CYP2C19* allele *2 in heterozygosis (*1/*2) and homozygosis (*2/*2) is related to intermediate and poor metabolizers, respectively. The presence of these alleles influences the metabolism of CLB.^13^ Indeed, individuals carrying one or two copies of the defective*2 allele might develop markedly elevated steady‐state plasma concentrations of N‐clobazam and be at higher risk of ADRs. However, N‐clobazam was confirmed to have a broad safety margin with a wide interindividual pharmacokinetic variability caused by factors other than CYP2C19 enzyme.

The presence of loss‐of‐function alleles also affects the metabolism of PB,^14^ PHT,^15^ BRV,^16^ LCM,^17^ and likely CBD, although there are currently not sufficient supporting data.^18^ Furthermore, notwithstanding CYP2C19 is considered a minor metabolic pathway of VPA, the influence of CYP2C19*2 and CYP2C19*3 alleles on its plasma concentrations has been recently reported.^19^

The CYP2C19*17 allele is associated with increased enzymatic activity. However, the magnitude of effects is considerably smaller than has been reported with CYP2C19*2 and CYP2C19*3, albeit in opposite directions. The functional effects of CYP2C19*17 are unlikely to be clinically significant except for drugs with very narrow therapeutic windows.^20^

*ABCB1* encodes P‐glycoprotein (Pgp), which is involved in the transport of most of the ASMs. The most convincing evidence for an association between *ABCB1* genotype and Pgp expression, function, and therapeutic drug response was reported by Loscher et al.,^21^ who studied in a prospective fashion whether the C3435T polymorphism affects the brain uptake of PB in patients with generalized epilepsy. This study seems to confirm the association between the CC genotype at *ABCB1* 3435 and ASM resistance described by Siddiqui et al.^22^ Although ABCB1 story was not replicated in most other studies and results obtained in ethnically different populations have been so far contradictory.^23^

*EPHX1* is involved in the transformation of CBZ‐10,11‐epoxide (CBZ‐E), the major CBZ metabolite, in CBZ‐10,11‐diol, that is inactive. In vitro expression studies of the *EPHX1* gene have shown that the 337T>C variant confers a 40% decrease in hydrolase activity, but in vivo conclusions are contrasting.^24^ *EPHX1 337CC*, which seems to reduce the efficacy of CBZ, was genotyped in six patients of our cohort. In one case (#2) test results confirmed that the current treatment was the optimal choice, whereas they could have avoided a previous therapeutic failure in another one (#21). Additionally, genotypic testing identified patients in whom CBZ should be avoided in the future.

In brief, genotypic testing provides additional information which might help clinicians to avoid therapeutic failures and ADRs, refining therapeutic choices, especially in patients who start treatment or are drug resistant. The availability of a noninvasive and safe test suggesting tailored treatments might be useful also in patients with comorbidities and polytherapy or in other special conditions like pregnancy.^25^ In this light, it is desirable to have a panel more specific for epilepsy, which may identify the polymorphisms of other enzymes involved in ASMs metabolism, such as the uridine 5′‐diphospho‐glucuronosyltransferases (metabolizing, among others, lamotrigine). However, these conclusions must be confirmed in larger studies.

The patient's cohort heterogeneity and small sample size can represent a limit for this study. A stratification of patients for age (<12 and >12 years) is recommended due to the immaturity of metabolic enzymes and drug transporters in children. Weight and plasmatic concentrations of ASMs before the test might be useful information to complete and confirm our results. A long‐term follow‐up (1 year at least) to monitor the response to the modified therapy according to test results, in terms of seizure control, and the onset of ASMs side effects is likewise needed. Finally, a limit may be found in the genotyping‐based approach. Pharmacogenetic research is currently ongoing. In silico targeted genotyping panels have long been the main tool for these studies, and the interlaboratory variability has been only partially reduced by the minimum list of alleles to be included in clinical testing provided by the Association for Molecular Pathology (AMP).^26^ Increased knowledge on drugs kinetics and advent massive parallel sequencing‐based approaches with open re‐analysis possibility will certainly shed light to this field in the next few years.

## 5 CONCLUSIONS

We suggest the use of an easy and safe pharmacogenomic testing on saliva may provide potential benefit in the clinical management of epilepsy patients when it is performed in newly diagnosed and drug‐resistant patients, as well as in those with comorbidities.

This approach could reduce direct and indirect costs due to lower failing treatments and ADRs incidence, in addition to an increased quality of life of patients. The use of genetic testing to guide epilepsy treatment is likely to increase in the future, as better understanding of the function of epilepsy genes will allow the application of precision medicine targeting the biological mechanisms responsible for epilepsy.

## AUTHOR CONTRIBUTIONS

PS contributed to the study conception and design. AR, GD'O, and RR drafted the manuscript. MSV, EA, VDG, CC, NS, NP, MT, GA, SC, CM, PR, PV, ER, and FZ were involved in the clinical care of the patients and in collecting clinical and genetic data. All authors have critically revised the manuscript and approved the final one as submitted.

## CONFLICT OF INTEREST STATEMENT

A.R has received honoraria from Kolfarma s.r.l, Proveca Pharma Ltd, and PTC Therapeutics. P. S. has served on a scientific advisory board for the Italian Agency of the Drug (AIFA); has received an unrestricted grant and congress support from FbHealth; has received honoraria from GW pharma, Kolfarma s.r.l., Proveca Pharma Ltd, and Eisai Inc.; and has received research support from the Italian Ministry of Health and Fondazione San Paolo. E.R. has received speaker fees or fundings or has participated in advisory boards for AIFA, Angelini, Arvelle Therapeutics, Eisai, GW Pharmaceuticals, Italian Ministry of Health, Italian Ministry of Research, Lundbeck, Pfizer, Kolfarma, UCB. All the other authors do not report conflict of interest.

## ETHICAL APPROVAL

The study was conducted according to the guidelines of the Declaration of Helsinki and approved by the Ethical Committee of the Gaslini Children's Hospital (Genoa, Italy) and the other participating centres. We confirm that we have read the Journal's position on issues involved in ethical publication and affirm that this report is consistent with those guidelines.

## INFORMED CONSENT STATEMENT

Informed consent was obtained from all subjects involved in the study.

## Supporting information


Table S1–S3

## Data Availability

All data generated or analyzed during this study are included in this published article [and its Additional information files]. Further inquiries can be directed to the corresponding author/s.
