# Supplementary material for: A real‐life pilot study of the clinical application of pharmacogenomics testing on saliva in epilepsy
Source: Epilepsia Open. 2023 May 15;8(3):1142–50. doi: 10.1002/epi4.12717 (PMC10472391; doi:10.1002/epi4.12717)
Supplement: Supplementary file 1 — Table S1–S3 [file EPI4-8-1142-s001.docx]

| **Supplementary Table 1. Mutant alleles and related SNPs of the analyzed genes.** | | |
| --- | --- | --- |
| **Gene** | **Mutant allele** | **Single nucleotide polymorphism** |
| **CYP1A2** | *****1F 163C>A | rs762551 |
| **CYP2C9** | **2* 430C>T | rs1799853 |
|  | **3* 1075A>C | rs1057910 |
| **CYP2C19** | **2* 681G>A | rs4244285 |
|  | **17* -806C>T | rs12248560 |
| **EPHX1** | 337T>C | rs1051740 |
| **ABCB1** | 3489+80C>T | rs2235048** |
| **used as proxy for 3435 C>T | | |

| **Supplementary Table 2. Genotype influence on drug response according to pharmacokinetic characteristics.** | | | | | | | |
| --- | --- | --- | --- | --- | --- | --- | --- |
|  | **Main metabolic route** | **P-Gp substrate** | ***CYP1A2***  *1F/*1F (FM) | ***CYP2C9***  *1/*2 (IM)  *1/*3 (IM)  *2/*3 (PM) | ***CYP2C19***  *1/*17 (FM)  *1/*2 (IM)  *2/*2 (PM) | ***EPHX1***  337T>C (CC)  ↓ CBZ efficacy | ***ABCB1***  3489+80C>T (CC)  Drug-resistant |
| Brivaracetam | Hydrolysis, CYP2C19 | No |  |  | *(FM)  ***§*** (IM, PM) |  |  |
| Cannabidiol | CYP2C19  CYP3A4  UGTs | Yes  (7-COOH-CBD) |  |  | *(FM)  ***§*** (IM, PM) |  | * |
| Cenobamate | UGTs  CYP2E1, CYP2A6, CYP2B6 | No |  |  |  |  |  |
| Carbamazepine | CYP3A4 | No |  |  |  | * |  |
| Clobazam | CYP3A4  CYP2C19 | Yes |  |  | *(FM)  ***§*** (IM, PM) |  | * |
| Clonazepam | CYP3A4 | Yes |  |  |  |  | * |
| Eslicarbazepine | UGTs | Yes |  |  |  |  | * |
| Ethosuximide | CYP3A4 | No |  |  |  |  |  |
| Everolimus | CYP3A4 | Yes |  |  |  |  | * |
| Felbamate | CYP3A4 | Yes |  |  |  |  | * |
| Gabapentin | Renal excretion | No |  |  |  |  |  |
| Lacosamide | CYP3A4  CYP2C9  CYP2C19 | No |  | ***§*** | *(FM)  ***§*** (IM, PM) |  |  |
| Lamotrigine | UGT1A4 | Yes |  |  |  |  | * |
| Levetiracetam | Renal excretion | Yes |  |  |  |  | * |
| Oxcarbazepine | UGTs | Yes |  |  |  |  | * |
| Perampanel | CYP3A4 | No |  |  |  |  |  |
| Phenobarbital | CYP2C9  N-glucosidation | Yes |  | ***§*** |  |  | * |
| Phenytoin | CYP2C9  CYP2C19 | Yes |  | ***§*** | *(FM)  ***§*** (IM, PM) |  | * |
| Pregabalin | Renal excretion | No |  |  |  |  |  |
| Primidone | CYP2C9 | Yes |  | ***§*** |  |  | * |
| Rufinamide | Hydrolysis | No |  |  |  |  |  |
| Stiripentol | CYP1A2  CYP2C19  CYP3A4 | Yes | * |  | *(FM)  ***§*** (IM, PM) |  | * |
| Tiagabine | CYP3A4 | Yes |  |  |  |  | * |
| Topiramate | Not extensively metabolised (~20%)  glucuronidation, hydroxylation and hydrolysis | Yes |  |  |  |  | * |
| Valproic acid | CYP2C9  CYPs  UGTs | No |  | ***§*** |  |  |  |
| Vigabatrin | Renal excretion | No |  |  |  |  |  |
| Zonisamide | CYP3A4  Reduction  N-acetylation | No |  |  |  |  |  |
| *FM, fast metabolizer; IM, intermediate metabolizer; PM, poor metabolizer; orange= need of monitoring due to: *reduced chance of optimal response, §increased risk of adverse events; green= optimal safety and efficacy profile* | | | | | | | |

| **Supplementary Table 3. *CYP2B6, CYP2D6, CYP3A4* genotypes and phenotypes.** | | | | | | |
| --- | --- | --- | --- | --- | --- | --- |
| **Patient Code** | **CYP2B6** | | **CYP2D6** | | **CYP3A4** | |
|  | **Genotype** | **Phenotype** | **Genotype** | **Phenotype** | **Genotype** | **Phenotype** |
| **#1** | *1/*1 | EM | ***41/*41** | **IM** | *1/*1 | EM |
| **#2** | *1/*6 | EM | *1/*2 | EM | *1/*1 | EM |
| **#3** |  |  |  |  |  |  |
| **#4** |  |  |  |  |  |  |
| **#5** |  |  |  |  |  |  |
| **#6** |  |  |  |  |  |  |
| **#7** | *1/*6 | EM | *1/*1 | EM | *1/*1 | EM |
| **#8** | *1/*6 | EM | ***2/*4** | **IM** | *1/*1 | EM |
| **#9** | *1/*1 | EM | *2XN/*4 | EM | *1/*1 | EM |
| **#10** |  |  |  |  |  |  |
| **#11** | *1/*1 | EM | *1/*1 | EM | *1/*1 | EM |
| **#12** |  |  |  |  |  |  |
| **#13** | *1/*1 | EM | *1/*1 | EM | *1/*1 | EM |
| **#14** | *1/*6 | EM | *1/*1 | EM | *1/*1 | EM |
| **#15** | *1/*6 | EM | *1/*2 | EM | *1/*1 | EM |
| **#16** |  |  |  |  |  |  |
| **#17** |  |  |  |  |  |  |
| **#18** | *1/*1 | EM | ***2/*4** | **IM** | *1/*1 | EM |
| **#19** | *1/*1 | EM | ***4/*4** | **PM** | *1/*1 | EM |
| **#20** | *1/*6 | EM | *2/*41 | EM | *1/*1 | EM |
| **#21** | *1/*1 | EM | ***1/*5** | **IM** | *1/*1 | EM |
| *Legend: EM= extensive metabolizer (standard); FM= fast metabolizer; IM= intermediate metabolizer; PM= poor metabolizer* | | | | | | |
